# *Plasmodium yoelii *blood-stage primes macrophage-mediated innate immune response through modulation of toll-like receptor signalling

**DOI:** 10.1186/1475-2875-11-104

**Published:** 2012-04-01

**Authors:** Yong Fu, Yan Ding, Taoli Zhou, Xiaolan Fu, Wenyue Xu

**Affiliations:** 1 Department of Pathogenic Biology, Third Military Medical University, 30 Gaotanyan Zhengjie, Shapingba District, Chongqing 400038, People's Republic of China; 2 Institute of Immunology, PLA, Third Military Medical University, 30 Gaotanyan Zhengjie, Shapingba District, Chongqing 400038, People's Republic of China

**Keywords:** *Plasmodium yoelii*, Macrophage, Toll-like receptors

## Abstract

**Background:**

Toll-like receptors (TLRs) signalling is reported to be primed by the infection of human malaria parasite, *Plasmodium falciparum*. However, little is known about the regulation of macrophages TLR signalling by the infection of lethal or non-lethal strain of rodent malaria parasites.

**Methods:**

BALB/c mice were infected with non-lethal strain *Plasmodium yoelii *17XNL or lethal strain *P. yoelii *17XL. Peritoneal macrophages were isolated to study its immune response to pRBC lysate, and TLRs (TLR2, TLR4, and TLR9) agonists, and the expression of TLRs and intracellular signalling molecules were also investigated by flow cytometry and semi-quantitive RT-PCR.

**Results:**

The reactivity of peritoneal macrophages from the mice infected with lethal strain *P. y *17XL or non-lethal strain *P. y *17XNL were enhanced to pRBC lysate, and TLR2, TLR4, and TLR9 agonists at one, three and five days post-infection. Of all the tested TLRs, only TLR2 was up-regulated on peritoneal macrophages of mice infected with either strain. However, transcription of intracellular signalling molecules MyD88, IRAK-1, and TRAF-6 was significantly up-regulated in peritoneal macrophages from mice infected either with *P. yoelii *17XL or *P. yoelii *17XNL at one, three and five days post-infection. However, the enhanced TLRs response of macrophage from *P. yoelii *17XNL-infected mice persisted for a much longer time than that from *P. yoelii *17XL-infected mice.

**Conclusion:**

Both *P. yoelii *17XL and 17XNL strains could enhance the response of peritoneal macrophages to pRBC lysate and TLR agonists, through up-regulating the expression of TLR2 and intracellular signalling molecules MyD88, IRAK-1, and TRAF-6. In addition, prolonged high response of macrophage from *P. yoelii *17XNL-infected mice might be associated with the more efficiently controlling of *P. yoelii *17XNL growth in mice at early stage.

## Background

Malaria remains one of the most devastating diseases worldwide, with ~40% of the population at risk, and 200-300 million new cases each year, resulting in about one million deaths annually [1]. The causative agents of malaria are parasitic protozoa belonging to the genus *Plasmodium*. Except for the virulence of infected malaria parasite, presentation of clinical malaria is mainly dependent on the balance between pro- and anti-inflammatory responses against these parasites. Individuals who exhibit particularly weak immune responses often lead to uncontrolled parasitaemia. Thus, understanding the regulation mechanism of the immune response brought on by infection with *Plasmodium *parasites will provide us with potential therapeutic approaches for treating infected individuals.

Although adaptive immune effectors, such as *Plasmodium*-specific CD4^+ ^αβ T cells and antibody [2], are mandatory for effective clearance of parasitized red blood cells (pRBCs) after infection, the control of parasite growth during the early stage of infection is largely dependent on the innate immune response. Previous study has shown that the primary peak of parasitaemia in T-cell-deficient mice tends to be comparable to that of wild-type mice [3]. It has been reported that NK cell-derived IFN-γ that contributes to the early control of *Plasmodium chabaudi *and *Plasmodium yoelii *infections [4,5]. However, a recent study showed that the macrophage-mediated innate immune response, but not IFN-γ, has a significant role in controlling the primary wave of *P. yoelii *infection [6]. Another study recently reported that a population of CD11b^high^Ly6C^+ ^monocyte migration from bone marrow to the spleen was important for killing of *P. chabaudi *blood stage at early stage [7].

It is well established that macrophages are activated by malaria parasites to release pro-inflammatory cytokines, mainly through toll-like receptors (TLRs). For example, *Plasmodium falciparum*-derived glycosylphosphatidylinositols (GPI) moieties are known to induce potent TNF responses in macrophages by TLR2, and to a lesser extent TLR4[8,9]. It has also been reported that *P. falciparum *haemozoin, a crystalline by-product of haemoglobin metabolism by malaria parasites, is recognized by TLR9 on macrophages[10], although more recently it has been suggested that instead haemozoin-bound nucleic acids are the true ligand for this receptor [11,12].

TLR signalling is subject to modulation by microorganisms. For instance, it is well known that lipopolysaccharide (LPS), the toxic wall component of Gram-negative bacteria, induces macrophages into a tolerance status by down-regulating TLR4 receptor expression [13], or its association with MyD88, and by IRAK-1 activation [14]. In contrast, infection of *P. falciparum *primes peripheral blood mononuclear cells (PBMC) for TLR signalling by enhancing mitogen-activated protein kinase (MAPK) activation during the early stage [15-17]. However, the infection of *P. yoelii *non-lethal strain 17XNL was reported to induce dendritic cell (DC) TLR tolerance during the late stage [18].

Little is known about the regulation of macrophages TLR signalling by the infection of lethal or non-lethal strain of rodent malaria parasites at the early stage. In the present study, both *P. yoelii *17XL and 17XNL strains were found to be able to enhance the response of peritoneal macrophages to pRBC lysate and TLR agonists, through up-regulating the expression of TLR2 and intracellular signalling molecules MyD88, IRAK-1, and TRAF-6.

## Methods

### Mice and *plasmodium*

BALB/c mice (specific pathogen free, ~6-8 week old females) were purchased from the Animal Institute of Third Military Medical University (Chongqing, China). These studies have been reviewed and approved by the Third Military Medical University Institute of Medical Research Animal Ethics Committee. *P. yoelii *17XNL is a non-lethal strain, originally isolated in 1965 from the blood of a wild thicket rat, *Thamnomys rutilans *[19]*, and P. yoelii *17XL is a lethal strain cloned from *P. yoelii *17XNL, which was suddenly virulent in the laboratory of J. Finerty [20]. Cohorts of 10 mice were infected intraperitoneally with 2 × 10^5 ^non-lethal strain *P. yoelii *17XNL-infected pRBCs or 2 × 10^5 ^lethal strain *P. yoelii *17XL. For control purposes, 10 mice were also infected with 2 × 10^5 ^RBCs taken from normal mice (nRBCs).

### Reagents and antibody

Biotin anti-mouse TLR4 (MTS510), TLR2 (6 C2 clone), TLR9 (M9.D6 clone), Biotin rat IgG2a and IgG2b isotype control antibodies, anti-mouse CD16/32 (93 clone), FITC-F4/80 and PE-streptavidin were all purchased from eBioscience (San Diego, CA). TLR2 agonist Pam3CSK4, TLR4 agonist LPS (*Escherichia.coli *0111:B4), TLR9 agonist CpG(ODN 1826), and its control CpG were purchased from InvivoGen (San Diego, CA).

### Isolation of peritoneal macrophage

All the mice infected with *P. yoelii *17XL or 17XNL, or injected with nRBCs, were administrated with 1 mg/ml thioglycollate (Sigma, St. Louis, MO, USA) via intraperitoneally three days before killing. Peritoneal exudate cells (PECs) were then extracted and allowed to adhere on tissue culture dishes for two hours, and non-adherent cells were removed. The adherent cells were collected as peritoneal macrophages, and F4/80 expression was analysed using a FACSCalibur flow cytometer with Cell Quest software (Becton Dickinson, Lincoln Park, NJ).

### Cytokine detection by ELISA

Peritoneal macrophage from mice infected with *P. yoelii *17XL, 17XNL, or nRBCs were cultured in the presence of 1 × 10^7 ^nRBC or pRBC lysate, or LPS (10 μg/ml), Pam3CSK4 (10 μg/ml), or CpG (10 μg/ml). Lysates from 1 × 10^7 ^red blood cells (RBCs) or pRBCs were prepared by twice freeze-thaw. After 24 hours, supernatants were collected and analysed by ELISA (eBioscience) according to manufacturer's instructions to detect IL-6 and TNF.

### Flow cytometry assay

Peritoneal macrophage were isolated from mice infected with *P. yoelii *17XL or 17XNL, or from mice injected with nRBCs, at one, three and five days after infection. 1 × 10^6 ^peritoneal macrophages were blocked with anti-mouse CD16/32 by incubation on ice for 30 min, then labelled with biotin-conjugated anti-mouse TLR4 and TLR2 for 30 min, followed by incubation with PE-streptavidin for 30 min. To examine TLR9 expression, peritoneal macrophages were permeabilized with fixation/permeabilization (eBioscience) prior to labelling with biotin-conjugated anti-mouse TLR9 and PE-streptavidin. Cells were finally analysed using a FACSCalibur flow cytometre using Cell Quest software (Becton Dickinson).

### Semi-quantitive reverse-transcriptase PCR

Total RNA was isolated from 1 × 10^6 ^peritoneal macrophages from mice injected with *P. yoelii *17XL or 17XNL strain, or with nRBCs, one, three and five days after infection using Trizol (Boehringer Mannheim, Germany), and reversely transcribed with MMLV (Promega, Madison, WI, USA). Initial cDNAs amount for PCR amplification were normalized with GAPDH signal, which was optimized to be visible, but not saturated. Changes in the expression of intracellular signalling molecules TRAF-6, IRAK-1, and MyD88 were investigated in peritoneal macrophages from mice infected with or without malaria parasites. Specific primers used were as follows: IRAK-1_781_: 5'- GAACAGCTATCAAGGTTTCGTCA-3'; IRAK-1_1260_: 5'-ACCAGCAAGGGTCTCCAGTA-3'; MyD_2341_: 5'-CCCACTCGCAGTTTGTTG-3'; MyD_2569_: 5'-CTCCCAGTTCCTTTGTTTG-3'; TRAF_162_: 5'-GGGCTACGATGTGGAGTT-3'; TRAF_396_: 5'-TACCGTCAGGGAAAGAAT-3'.

### Statistic analysis

Statistical significance was determined with SPSS software (version 13.0). Differences between two experimental groups (*P.yoelii *17XNL-infection vs naive or *P.yoelii *17XL-infection vs naive), or two time points were analysed for statistical significance by means of a nonparametric Mann-Whitney U test, and for multiple groups (TLR2 expression among *P.yoelii *17XNL-infected, *P.yoelii *17XL-infected and naive mice) using Kruskal-Wallis test. *P <0.05 or **P <0.01 were considered statistically significant.

## Results

### *Plasmodium yoelii *17XNL and 17XL infection enhance the response of peritoneal macrophage to pRBCs

**Figure 1:**
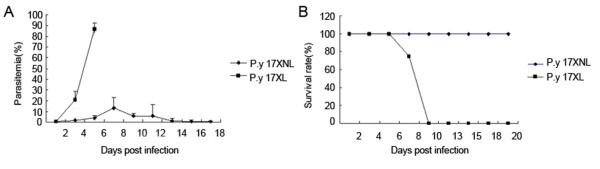
***In vivo *infection course after inoculation with *P. y *17XL or 17XNL**. BALB/c were intraperitoneally injected with 2 × 10^5 ^*P. yoelii *17XL or 17XNL parasites. Parasitaemia was recorded every other day, respectively.

*Plasmodium yoelii *17XNL is a non-lethal *Plasmodium *strain, which grows slowly and would be cleared by mice at 20 days after infection. In contrast, *P. yoelii *17XL is a lethal strain, which grows quickly and infected mice usually die at six to nine days after injection (Figure 1).

**Figure 2:**
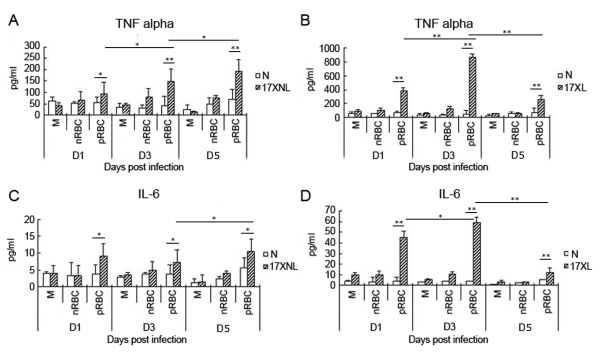
**Peritoneal macrophage response from mice infected with *P. y *17XL or 17XNL to pRBC lysate**. Three days after mice were treated with thioglycollate, peritoneal macrophages were isolated from mice on one, three and five days after intraperitoneally injected with 2 × 10^5 ^nRBCs (N), 2 × 10^5 ^*P. yoelii *17XNL- or 17XL-infected RBCs. Each 1 × 10^6 ^macrophages from mice infected with *P. yoelii *17XNL were stimulated with medium (M), 1 × 10^7 ^*P. yoelii *17XNL-infected RBCs lysate for 24 h, and supernatants were collected to detect TNF-α (A) and IL-6 (C). Each 1 × 10^6 ^macrophages from mice infected with *P. yoelii *17XL were also stimulated with medium (M), 1 × 10^7 ^*P. yoelii *17XL-infected RBCs lysate for 24 h, and supernatants were collected to detect TNF-α (B) and IL-6 (D). Three individual experiments were performed, and all data are presented as the mean ± SD (*P <0.05, **P <0.01).

Since the macrophage-mediated innate immune response plays an important role in controlling the primary wave of *P. yoelii *infection [6], the responsiveness of macrophages collected on one, three and five days after infection with either the *P. yoelii *lethal 17XL strain or non-lethal 17XNL strain was investigated. Pooled adherent PECs were used as peritoneal macrophages in the following experiments without additional indication, as 85% of pooled adherent PECs were positive for the macrophage-specific marker F4/80(data not shown). Compared to the peritoneal macrophages from mice injected with nRBCs, the reactivity of macrophages from mice infected either with *P. yoelii *17XL or 17XNL was enhanced at one, three and five days post infection, as both inflammatory cytokines TNF and IL-6 released by macrophages stimulated with pRBC lysate were much higher in either strain infected mice (Figure 2). However, the response of macrophages reached a peak at three days, and it was then tend to return to the baseline level at five days post *P. yoelii *17XL-infection (Figure 2B,D). In contrast, the response of macrophages from *P. yoelii *17XNL-infected mice even increased at five days post infection (Figure 2A,C). Thus, these data suggested that the response of peritoneal macrophage was enhanced after mice were infected either with *P. yoelii *17XL or 17XNL strain, but the duration time was much longer for the macrophages from *P. yoelii *17XNL-infected mice.

### *Plasmodium yoelii *17XNL and 17XL infection prime the response of peritoneal macrophages to TLR agonists

**Figure 3:**
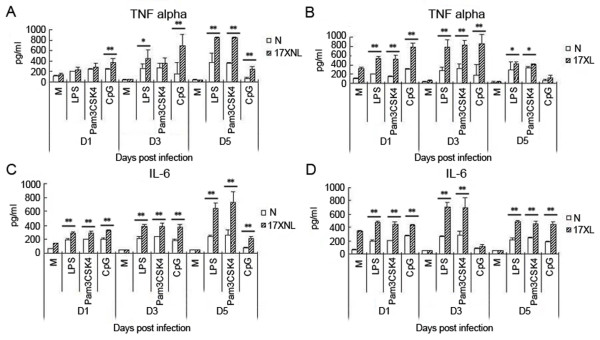
**The response of macrophages from mice infected with *P. y *17XL or 17XNL to TLR agonists**. Peritoneal macrophages were collected from mice at one, three and five days after being intraperitoneally injected with 2 × 10^5 ^nRBCs (N), 2 × 10^5 ^*P. yoelii *17XL- or 17XNL-infected RBCs. 1 × 10^6 ^macrophages were then stimulated with medium (M), LPS (10 μg/ml), Pam3CSK4 (10 μg/ml), or CpG (10 μg/ml) for 24 h, and cell supernatants were collected to detect TNF-α (A) and IL-6 levels (B). Three individual experiments were performed, and pooled data are presented as the mean ± SD (*P <0.05, **P <0.01).

It is well known that malaria parasite components, including GPI and haemozoin, could induce macrophages to release inflammatory cytokines through TLR2/4 and TLR9[8-10]. To investigate the mechanism of the enhanced response of macrophages to pRBC lysate primed by infection of *P. yoelii *17XL or 17XNL, the levels of inflammatory cytokines of TNF and IL-6 released by macrophages stimulated with TLR2 agonist Pam3CSK4, TLR4 agonist LPS, or TLR9 agonist CpG, were measured. As shown in Figure 3, peritoneal macrophages stimulated with any of the three TLR agonists exhibited dramatically increased TNF and IL-6 levels at one, three and five days after infection with *P. yoelii *17XNL(Figure 3A,C) or 17XL(Figure 3B,D), which was consistent with the response of macrophages to pRBC lysate. Take together, these data supported that the response of macrophages primed by *P. yoelii *17XL or 17XNL infection was due to enhanced TLR response of macrophages.

### Effect of *P. Yoelii *17XL and 17XNL infection on TLR expression of peritoneal macrophage

**Figure 4:**
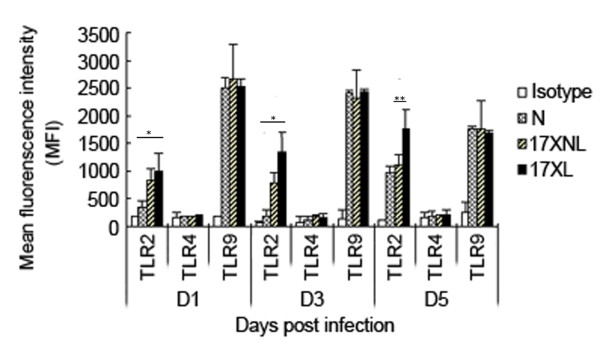
**TLR2, TLR4 and TLR9 expression on macrophages from mice infected with *P. y *17XL or 17XNL**. Peritoneal macrophages were collected from mice at one, three and five days after they had been intraperitoneally injected with nRBCs, *P. yoelii *17XL- or 17XNL-infected RBCs. Macrophages were labelled with anti-mouse TLR4, TLR2 and TLR9, and analysed on a FACSCalibur using Cell Quest software. Data are presented as the mean fluorescence intensity (MFI), and values are shown as mean ± SD (*P <0.05, **P <0.01).

It was previously reported that TLR4 and MD-2 over-expression was attributed to TLR4 signal priming induced by *Propionibacterium acnes *[21]. Furthermore, *P. falciparum*-induced priming of monocyte TLR response is also associated with increased expression of both TLR2 and TLR4 [15]. Hence, the expression of TLR2, TLR4, and TLR9 on peritoneal macrophages from mice infected with *P. yoelii *17XL or 17XNL was investigated to explore the mechanism of TLR response priming induced by *P. yoelii*. As shown in Figure 4, macrophages from mice infected with 17XL or 17XNL had significantly increased expression of TLR2, but not TLR4 or TLR9, compared to the control mice at all time points measured. The strong response of macrophages from *P. yoelii *17XL- or 17XNL-infected mice to TLR2 agonist Pam3CSK4 might be correlated with the relatively high level of TLR2 on PECs.

### Infection of *P. Yoelii *17XL and 17XNL up-regulate TLR-MyD88 dependent pathway intracellular molecules

**Figure 5:**
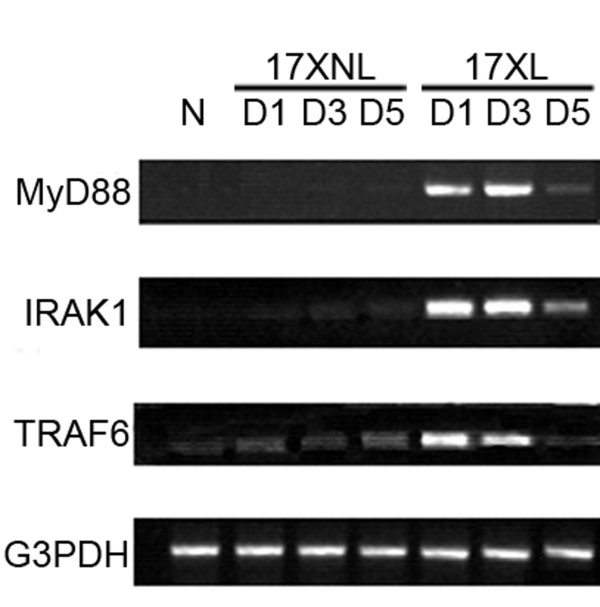
**Semi-quantitative RT-PCR analysis of TLR intracellular molecules in macrophages from mice infected with *P. y *17XL or 17XNL**. Peritoneal macrophages were collected from mice at one, three and five days after they were intraperitoneally injected with nRBCs, *P. yoelii *17XL- or 17XNL-infected RBCs. Total RNA was then isolated from the peritoneal macrophages, and the intracellular molecules, MyD88, IRAK1, and TRAF-6 were amplified, with G3PDH signal used as an internal control (N = 3; one representative experiment was shown).

These data so far does not explain the underlying mechanism of the primed TLR4 and TLR9 responses of macrophages induced by *P. yoelii *17XL and 17XNL infection[15]. Since p38 activity enhancement are related to TLR response priming in malaria parasite infection [16], then the transcription of MyD88, IRAK-1, and TRAF6, which are shared by the TLR2-, TLR4-, and TLR9- mediated MyD88-dependent pathway, were investigated in macrophages from *P. yoelii *17XL- or 17XNL-infected mice. As shown in Figure 5, the transcriptional levels of MyD88, IRAK-1, and TRAF-6 were significantly increased in mice infected with either *P. yoelii *17XL or 17XNL compared to control mice at one, three and five days post-infection. The change pattern of transcription levels of MyD88, IRAK-1, and TRAF-6 of macrophage was consistent with its response to pRBC lysate after infection with either strain. These data strongly suggested an important role for the up-regulation of MyD88, IRAK-1, and TRAF-6 in the TLR response priming induced by either *P. yoelii *strain infection.

## Discussion

Macrophage-mediated innate immune response is critical for controlling the *P. yoelii *parasitaemia at its early stage, so it tends to be modulated by exposure to malaria parasites. It was previously reported that *Plasmodium berghei *infection inhibits IL-12 p40 production by peritoneal macrophages at the transcriptional level [22]. However, infection with lethal strain *P. yoelii *17XL or non-lethal strain *P. yoelii *17XNL was found to be able to prime the response of macrophages through up-regulating TLR2 expression and signalling intracellular molecules, although infection of the two strains resulted in dramatically different disease outcome.

Pre-exposure to a variety of TLR agonists, including LPS, Pam3CSK4, and CpG, often induces macrophages into a tolerant status to prevent over-activation [23-25]. It is well known that pre-administration of a low dose (sub-lethal dose) of LPS induces macrophages into endotoxin tolerance to protect the host from the challenge of lethal-dose of LPS. However, tissue injury[26] and *Propionibacterium *infection [21] have been demonstrated to prime the TLR response. In this study, the lethal *P. yoelii *17XL strain was also found to enhance the response of murine peritoneal macrophages to pRBC at one, there and five days post-infection (Figure 3B-D), which is consistent with recent reports of priming the TLR response with *P. f *at the early stage [15-17]. Interestingly, infection with the non-lethal *P. yoelii *17XNL strain could also prime the response of macrophages to pRBC lysate (Figure 3A-C). Like the response of macrophages to pRBC lysate, macrophages from *P. yoelii *17XL- or 17XNL-infected mice responded to TLR2, TLR4, and TLR9 agonists much more strongly than macrophages from nRBCs-injected mice at one and there days post-infection (Figure 4). Thus, the increased response of macrophages to TLR2, TLR4, and TLR9 agonists resulted in their hypersensitivity to pRBC lysate.

In a previous study, McCall *et al *attempted to correlate TLR2/4 expression with the priming response, however the investigators did not observe enhanced expression of TLR2/4 on PBMCs [17]. In contrast, Flanklin *et al *found that TLR2, TLR4 and TLR9 expression was significantly augmented in PBMCs from patients with relatively high parasitaemia [15]. Here, the infection with *P. yoelii *17XL or 17XNL induced the expression of TLR2, but not TLR4 and TLR9, on murine macrophages in the present study (Figure 5). The disparity could be interpreted as *P. yoelii *used in this study, but *P.f *was used in their research. Interestingly, the transcription levels of intracellular molecules of the MyD88-dependent pathway were also found to be augmented in macrophages from *P. yoelii *17XL- or 17XNL-infected mice (Figure 5). Therefore, hypersensitivity of macrophages to TLR agonists was contributed to up-regulation of intracellular signalling molecules by malaria parasite infection. However, it remains to be determined whether up-regulation of MyD88, IRAK-1, and TRAF-6 would result in enhancement of MAPK activation, which was previously contributed to priming the TLR response on PBMCs from *P. f-*infected patients [16].

It was recently reported that malaria-induced priming of the TLR response was TLR9-, MyD88-, and IFN-γ-dependent[15]. Hence, it is reasonable to find that lethal and non-lethal strains can prime the macrophage response in this study, as a relative high level of IFN-γ is induced in the spleen of either strain-infected mice during the early stage [27]. It is well known that IFN-γ was mainly secreted by NK and T cells after infection with lethal strain *P. yoelii *17XNL and nonlethal strain *P. yoelii *17XL [4], but the production of IFN-γ by NK cells required the help of IL-12 of DC activated by rodent malaria parasite [28].

Single amino acid substitution of erythrocytic binding ligand (EBL) was reported to determine the erythrocyte invasion preference and virulence of *P. yoelii *17XL and *P. yoelii *17XNL [29], but the early induction of TGF-β[30] and activation of CD4^+^CD25^+^T cells could suppress the host immune response, and result in the overgrowth of *P. yoelii *17XL in mice. Although the level of macrophage response between the two strains could not be compared in this study, as their parasitaemia were significantly different at one, there and five days post infection (Figure 1), the duration time of primed TLRs response of macrophage from *P. yoelii *17XNL-infected mice was much longer than that from *P. yoelii *17XL-infected mice. This is consistent with a relative higher level of IFN-γ in the spleen of *P. yoelii *17XNL-infected mice than that of *P. yoelii *17XL-infected mice [27], and might be associated with more efficiently controlling of *P. yoelii *17XNL growth than *P. yoelii *17XL in mice at the early stage.

## Conclusion

It was observed that *P. y*, either its lethal 17XL strain or non-lethal 17XNL strain, primes the TLR response on macrophages, mainly through modulating the transcription of intracellular signalling molecules. However, the enhanced macrophage response induced by *P. yoelii *17XNL maintained longer than that induced by lethal *P. yoelii *17XL. This finding provides us with a novel aspect of TLR response modulated by malaria parasites with different virulence, and clues to understand the control mechanism of the primary wave of *Plasmodium yoelii*.

## Abbreviations

DC: Dendritic cell; EBL: Erythrocytic binding ligand; GPI: Glycosylphosphatidylinositols; LPS: Lipopolysaccharide; MFI: Mean fluorescence intensity; PBMC: Peripheral blood mononuclear cells; MAPK: Mitogen-activated protein kinase; RBCs: Red blood cells; nRBCs: Normal red blood cells; PECs: Peritoneal exudate cells; pRBCs: Parasitized red blood cells; *P. chabaudi*: *Plasmodium chabaudi; P. yoelii*: *Plasmodium yoelii; P. falciparum*: *Plasmodium falciparum*; TLRs: Toll-like receptors.

## Competing interests

The authors declare that they have no competing interests.

## Authors' contributions

WYX designed research; YF, YD, TLZ, XLF performed research; WYX analysed data and wrote paper. All authors have read and approved the final manuscript.
